# Case Series of Incidentally Found Chronic Airway Foreign Bodies: A Single‐Center Experience

**DOI:** 10.1155/crpu/8374435

**Published:** 2026-04-29

**Authors:** Mwanaada Kilima, Hari Kishan Gonuguntla, Belgundi Preeti Vidyasagar, Syed Shabbir Hussain, Sibtain Moledina

**Affiliations:** ^1^ Division of Interventional Pulmonology, Yashoda Super Specialty Hospitals Secunderabad, Secunderabad, India; ^2^ Department of Internal Medicine, Muhimbili National Hospital, Dar es Salaam, Tanzania, mnh.or.tz; ^3^ Department of Internal Medicine, Muhimbili University of Health and Allied Sciences, Dar es Salaam, Tanzania, muchs.ac.tz

**Keywords:** chronic airway foreign body, flexible bronchoscopy, foreign body retrieval, interventional pulmonology

## Abstract

Foreign body aspiration among adults and older children could be more common than previously documented. We present below eight cases of incidentally found airway foreign bodies (FB) during endoscopic evaluation of patients performed due to various indications such as chronic cough, nonresolving pneumonia and suspected malignancy, among others. A high index of suspicion and prompt management are required to prevent morbidity and complications associated with long‐standing airway FB.

## 1. Introduction

Foreign body aspiration is a serious, potentially life‐threatening medical condition which occurs more frequently in children [1]. Although adults seldom aspirate foreign bodies (FB), it is relatively more of a diagnostic challenge [2, 3]. The duration of time between the aspiration incident and the onset or worsening of symptoms can vary from days to months, or even years [2, 3]. Prolonged indwelling FB is not only associated with a higher respiratory morbidity, but also poses a significant therapeutic challenge [4].

Our center sees around 20 cases of airway FB per year. In this series, we present eight cases of incidentally found chronic airway FBs at our center from 2022 to 2023. Clinical presentation, imaging and bronchoscopy findings are discussed. We also elaborate the therapeutic approaches used in the successful retrieval of the airway FBs. The characteristics of the cases are summarized in Table 1.

**Table 1:** Summary of the eight cases described.

Patient age (years)	Patient gender	Location of foreign body	Outcome
31	Male	Right lower lobe bronchus	FB removed
12	Male	Left lower lobe	FB removed
23	Male	Bronchus intermedius	FB removed
58	Male	Right lower lobe	FB removed
82	Male	Right lower lobe	FB removed
55	Male	Right main bronchus	FB removed
82	Male	Left main bronchus	FB removed
69	Male	Lingula	FB removed

### 1.1. Case 1

A 31‐year‐old male was referred to us for mediastinal lymph‐node biopsy, found on computed tomography (CT) of the chest. He gave a 5‐year history of worsening cough with mucopurulent expectoration, associated with shortness of breath. He had been repeatedly treated as pneumonia and also asthma, with minimal improvement.

**Figure 1:**
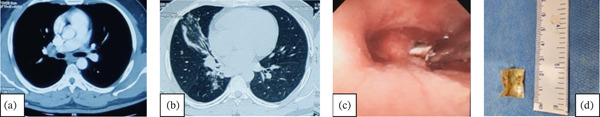
(a and b) CT chest showing right hilar and subcarinal mediastinal adenopathy and consolidation atelectasis of right middle lobe. (c) Foreign body embedded in dense granulation tissue. (d) Retrieved plastic foreign body with sharp edges.

A chest CT scan was evident for mediastinal lymphadenopathy (right hilar and subcarinal nodes), occlusion of the right lower lobe bronchus with consolidation atelectasis of the right middle lobe (Figure 1a,b). A fiberoptic bronchoscopic examination revealed narrowing of the right mainstem bronchus with dense whitish plaques and purulent secretions from both the right middle lobe and lower lobe. Following thorough bronchial toileting, a white flat object with sharp edges, apparently of plastic material, was visualized within dense granulation tissue in the right lower lobe bronchus (Figure 1c). On further discussion with the family, one family member recalled a history of choking in the patient about 11 years ago. He apparently aspirated a plastic sheath, which he had placed in his mouth while doing repair work on a ceiling fan. A chest radiograph was found to be normal and no further steps were taken thereafter.

Under general anesthesia, a rigid bronchial barrel was placed in right mainstem bronchus. Initial dis‐impaction with Fogarty balloon and multiple retrieval attempts using biopsy forceps, rat‐toothed forceps and basket by flexible bronchoscope failed as the FB remained tightly impacted. Application of several rigid forceps was then undertaken to achieve a firm grip of the FB. Rotatory maneuvers were employed to disimpact and successfully remove the FB under vision. It was a plastic sheath with sharp edges (Figure 1d). To rule out any coexistent or alternate etiological explanation for lymphadenopathy, endobronchial ultrasound (EBUS) guided transbronchial needle aspiration (TBNA) was also done which showed a reactive lymphadenopathy.

### 1.2. Case 2

A 12‐year‐old boy presented with complaints of cough and shortness of breath for a period of 6 months. There was a history of aspiration of a pen cap 6 months prior to the onset of symptoms. During the event, the child experienced a bout of severe cough. A chest radiograph was done following the incident and was found to be normal. Parents were therefore reassured that there was no airway foreign body. He was apparently asymptomatic until 6 months later when he sought medical advice once again due to recurrent cough. He was prescribed inhaled bronchodilators and corticosteroids for suspected asthma with no improvement. The patient therefore came to our facility for further evaluation and treatment.

**Figure 2:**
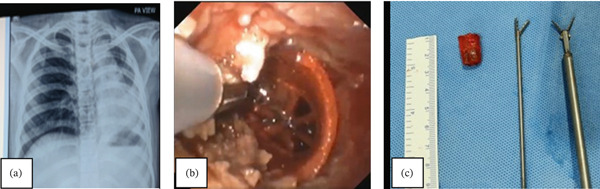
(a) Chest radiograph showing atelectasis of left lung. (b) Granulation removal using cryoablation. (c) Retrieved pen cap with rigid forceps.

Initial chest radiograph showed left lung atelectasis (Figure 2a). A chest CT suggested possibility of mucus plugging or granulation tissue in distal part of left mainstem bronchus; as well as consolidation with volume loss involving left lower lobe and left upper lobe.

Diagnostic bronchoscopy revealed stenosis and granulation tissue in the left main bronchus with a foreign body beneath it in the left lower lobe. Granulation tissue was ablated using electrosurgery and cryo‐application with repeated freeze‐thaw cycles. The procedure was done in two sittings. Endobronchial visualization of FB could only be achieved after adequate degranulation as described (Figure 2b). However, its retrieval remained quite challenging. Multiple attempts with flexible bronchoscopic tools failed. A rigid bronchial tube was used to mechanically dilate the area and pass beyond the stenosis. The FB was grasped with rigid forceps and extracted successfully. It was found to be a plastic pen cap. (Figure 2c).

### 1.3. Case 3

**Figure 3:**
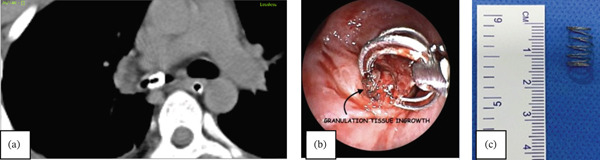
(a) Axial chest CT image showing a metal‐appearing FB in right intermedius bronchus. (b) Metallic foreign body embedded in granulation tissue (c) Retrieved metallic spring FB.

A 23‐year‐old male who worked as a mechanic was referred from a health center for evaluation of chronic cough of 6 months′ duration. Physical examination including respiratory system examination was unremarkable. Chest radiography showed presence of metallic opacity in retrocardiac area, which was apparently mistaken in earlier evaluation to be clothing artifact. This was more evident on chest CT imaging, which showed a metal‐appearing FB in right bronchus intermedius (Figure 3a). There was no report of prior history of aspiration.

Bronchoscopy showed the presence of a metallic foreign body in the bronchus intermedius tightly embedded in granulation tissue (Figure 3b). A taxing removal was achieved with flexible bronchoscopy forceps, preceded by repeated cryo‐application by Erbe 1.1‐mm probe. This was done via rigid bronchoscopy under general anesthesia. The inorganic FB was a metallic spring object (Figure 3c).

### 1.4. Case 4

**Figure 4:**
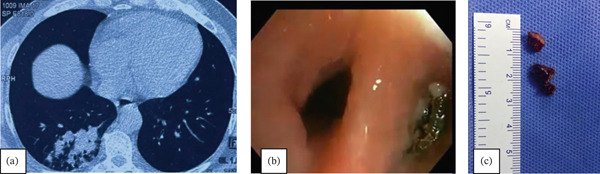
(a) CT Chest showing right lower lobe consolidation (b) Impacted foreign body in posterior basal segment of right lower lobe (c) Retrieved foreign bodies in three pieces.

A 58‐year‐old male presented with complaints of recurrent respiratory tract infection for 12 months. At his primary contact physician, a chest CT scan had been done on three occasions which showed right lower lobe consolidation with no sign of resolution despite antibiotic use (Figure 4a).

A diagnostic bronchoscopy at our facility showed an impacted foreign body in the right lower lobe posterior basal segmental bronchus—RB10 (Figure 4b). The patient could not recall an episode of choking. However, he admitted to having a habit of betel nut chewing during bedtime. Successful retrieval of FB in three pieces was achieved by initial disimpaction using Fogarty balloon inflations followed by cryo‐extraction with a 1.1‐mm Erbe probe via flexible bronchoscopy. An 11‐mm rigid bronchoscope barrel was used as a conduit under general anesthesia.

### 1.5. Case 5

**Figure 5:**
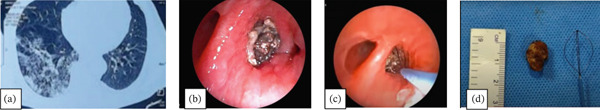
(a) Chest CT showing consolidation involving right posterior and lateral basal segments. (b) Organic foreign body in right lower lobe bronchus. (c) FB in dormia basket. (d) Retrieved FB with dormia basket.

An 82‐year‐old male patient was referred for further evaluation due to nonresolving pneumonia. He had complaints of cough with expectoration for 4 months associated with fever and shortness of breath. Evaluation with Chest CT showed consolidatory changes involving right posterior and lateral basal segments (Figure 5a).

On bronchoscopy, there was an organic foreign body in the right lower lobe bronchus (Figure 5b). Under general anesthesia via laryngeal mask airway, a flexible bronchoscope was used for extraction of the FB. Using a 4‐mm Fogarty balloon, the FB was extracted and successfully removed using a dormia basket (Figure 5c). The FB was a piece of meat, measuring 15 × 10 mm (Figure 5d). There were no complications and the patient fared well after the procedure. He was also astonished to have had a piece of meat in the airway as he could not recollect the occurrence of any incidence of choking prior.

### 1.6. Case 6

**Figure 6:**
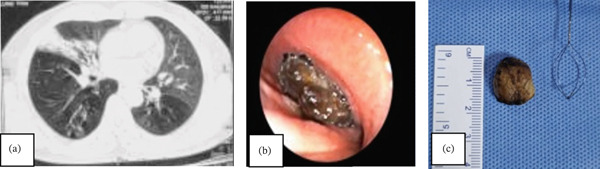
(a) Chest CT showing consolidation‐atelectasis of right middle lobe. (b) Deeply impacted FB at the origin of right main bronchus. (c) Retrieved FB (hog plum nut) with dormia basket.

A 55‐year‐old man was referred to our center for bronchoscopic examination to evaluate nonresolving pneumonia. As evidenced by a chest CT imaging, there was a consolidation atelectasis of the right middle lobe (Figure 6a).

Flexible bronchoscopic examination showed a deeply impacted foreign body with intense inflammation around it at the origin of the right main bronchus (Figure 6b). Using a Fogarty balloon catheter, the foreign body was dislodged by inflating the balloon distally. Using a basket, the FB was successfully extracted from the airways. The procedure was done using a flexible bronchoscope via rigid bronchoscope tracheal barrel as a conduit under general anesthesia. The FB was identified to be a nut (Figure 6c). Upon inspection, the outer shell of the nut was adhered to the bronchial mucosa, which was carefully extracted using a 1.7‐mm Erbe cryoprobe.

The family was approached after the procedure and they reported that the patient had a habit of chewing Indian hog plums, which is one of the three rejuvenating herbs that constitute the Triphala formulation used for treatment of cough and other medical ailments as per Indian herbal medicine.

### 1.7. Case 7

An 82‐year‐old male patient, smoker with 30 pack years was referred to our facility for evaluation of right upper lobe nodule. He presented at a peripheral hospital with complaints of cough for a period of 1 month.

**Figure 7:**
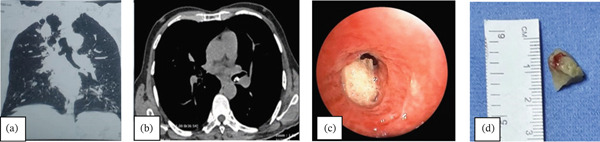
(a and b) Chest CT showing radio‐opaque FB in left main stem bronchus. (c) Pale‐yellow FB in left main bronchus with bony appearance. (d) Retrieved chicken bone.

Posteroanterior chest radiograph showed a round opacity in the right upper zone. A contrast chest CT was done for suspicion of malignancy. It revealed a right upper lobe nodular lesion with speculated margins; enlarged subcarinal and hilar lymph nodes; and a tree‐in‐bud nodular appearance was present in the left lower lobe. A clear radio‐opaque foreign body in the left main bronchus was also perceived (Figure 7a,b).

During diagnostic bronchoscopy, a hard pale‐yellow substance with a bony appearance was visualized in the left mainstem bronchus (Figure 7c). Using rat‐toothed forceps via a flexible bronchoscope, the FB was clamped and withdrawn out of the airway. The FB was seemingly a chicken bone (Figure 7d). The presence of an airway foreign body was as much of a surprise for the patient as it was for our team. Radial EBUS‐guided cryo biopsy of the right upper lobe nodule was also performed in the same sitting. The histopathological examination revealed lung adenocarcinoma.

### 1.8. Case 8

**Figure 8:**
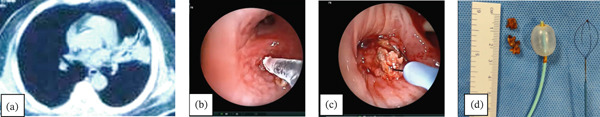
(a) Chest CT showing consolidation in lingular segment of left upper lobe. (b) Degranulation using cryo probe. (c) FB in dormia basket. (d) Retrieved meat pieces with Fogarty balloon and dormia basket.

A 69‐year‐old male presented with a 2‐week history of productive cough and fever associated with shortness of breath. Evaluation with thoracic CT scan showed consolidation with air bronchogram involving the lingula segment of the left upper lobe (Figure 8a). He was treated with antibiotics without improvement.

A foreign body with granulation tissue was visualized in the lingula during bronchoscopic examination. It was proximally displaced using a Fogarty balloon and extracted in three pieces with a Dormia basket (Figure 8c), after cryo‐degranulation (Figure 8b). Balloon dilatation and cryoablation were done following the FB extraction. This was a flexible bronchoscopy procedure, via a rigid tracheal barrel as a conduit, under general anesthesia. The FB was a piece of meat (Figure 8d) for which the patient could not recall the circumstances of its aspiration.

## 2. Discussion

This case series has put forward a number of unusual scenarios of chronic airway FB. This is due to delayed presentation and incidental finding after aspiration of objects, some of which were as large as meat pieces, nuts, and chicken bone. Our first patient′s FB, for instance, had been lodging in the bronchus and remained occult for a period of 11 years. Advanced age was the only apparent risk factor for FB aspiration among two of our patients. Other risk factors associated with FB aspiration, such as stroke, neuromuscular disorders, trauma, or seizures, could not be identified.

Symptoms of FB aspiration are variable depending on the degree and location of obstruction among other factors. The classic triad of coughing, wheezing, and diminished breath sounds is rare, especially among adults [5]. Nearly all our patients were adults. Moreover, the history of aspiration is not always obvious, leading to delayed recognition and treatment of airway FBs [5, 6]. Only two of our patients could recall an incidence of choking. However, despite a clear history of a witnessed FB aspiration in the two cases, the diagnosis of pneumonia and asthma had been repeatedly entertained. As a result, patients developed serious complications such as recurrent pneumonia, atelectasis, and bronchial stenosis, similar to previous reports of morbidity related to chronic airway FBs [6, 7]. These cases, among others reported in the literature, emphasize that foreign body aspiration should be considered in cases of prolonged respiratory symptoms [8–10]. Two of our patients were delayed based on normal chest radiography findings. It is worth noting that a chest x‐ray is not specific in diagnosing FB aspiration. In fact, a normal chest x‐ray does not exclude the presence of airway FB [11].

Airway foreign body removal is a challenging procedure. Failures and complications could arise due to a large foreign body that cannot be gripped, impacted FB in extensive granulation tissue, excessive tissue scarring, and sharp FB [12]. Prior preparations can be made if the FB is diagnosed earlier. In the cases of our patients, quick decision and planning had to be made amidst astonishment. On several occasions, extensive degranulation had to be done prior to the FB extraction.

Rigid bronchoscopy is the gold standard procedure for FB removal, especially among children [13]. However, flexible bronchoscopy is increasingly becoming the more preferred technique, particularly in adults [12, 14]. Flexible bronchoscope has better navigational properties permitting examination and manipulation of the lower airways with less trauma [15, 16]. The choice between flexible and rigid bronchoscopy should be guided by patient factors, FB characteristics, and operator expertise. Flexible bronchoscopy is generally preferred in adults due to its superior navigation of distal airways, lower risk of trauma, and ability to examine subsegmental bronchi under conscious sedation [1, 12, 14]. Rigid bronchoscopy, however, remains the gold standard in children, for large or sharp objects, or when significant bleeding, granulation tissue, or airway compromise is anticipated [1, 12, 13].

At our center, flexible bronchoscopy has been the modality of choice and has been used with great success as presented in the cases. Rigid bronchoscopic techniques were only employed on two occasions when the former failed. The use of cryotherapy has resulted in successful disimpaction of FBs deeply buried in granulation tissue. The flexible bronchoscopy procedures were performed using rigid bronchoscopes or laryngeal mask airway as a conduit. There were no complications and concomitant conditions such as pneumonia and airway stenosis were managed accordingly. Clinical, radiological and bronchoscopic follow‐up was regularly done for all patients.

The selection of retrieval devices also influences procedural success. Rat‐tooth forceps are effective for firm or irregularly shaped objects, such as bones or nuts, particularly in larger airways [12, 16]. Dormia baskets are useful for small, smooth, or difficult‐to‐grasp FBs, whereas Fogarty catheters can be threaded past loosely lodged or distal FBs to facilitate extraction [12, 16].

Adjunctive ablation techniques are employed depending on the nature of granulation tissue or airway obstruction. Cryoablation is particularly useful for chronic FBs deeply embedded in granulation tissue, allowing safe tissue debridement with minimal thermal injury [12]. Cauterization is preferred for highly vascular granulation tissue, both to control bleeding and to remove obstructing tissue [12]. Balloon dilation is reserved for cases with postimpaction bronchial stenosis, restoring airway patency after tissue removal [12]. At our center, these techniques were applied as needed, often in combination with flexible bronchoscopy, and contributed to successful FB removal without complications. Prior preparation and rapid decision‐making were essential, particularly in chronic cases where extensive degranulation was required before extraction [1, 4, 17].

In conclusion, airway FB may go unnoticed for many years. Clinicians ought to have a low threshold for suspicion when faced with cases of prolonged respiratory symptoms, whether or not a history of aspiration is reported. Relevant diagnostic work‐up should be done. Flexible bronchoscopy has shown great success in FB retrieval. Furthermore, utilization of cryo‐degranulation is a useful approach in facilitating removal, even for deeply impacted chronic airway FBs.

Though historical, one must not forget that it was the extraction of a chicken bone by Gustav Killian using primordial rigid bronchoscopy as a tool that has heralded the journey of interventions in the field of pulmonology.

## Author Contributions

M.K., H.K.G., B.P.V., S.H.S. were all involved in the management of these patients. M.K. wrote the initial draft of the manuscript. S.M. was significantly involved in writing and reviewing of the manuscript and follow up of any corrections in the manuscript. All authors have reviewed and agreed on the final manuscript.

## Funding

No funding was received for this manuscript.

## Ethics Statement

The authors declare that appropriate informed consent was obtained for the publication of this manuscript and accompanying images.

## Conflicts of Interest

The authors declare no conflicts of interest.

## Data Availability

Data sharing is not applicable to this article as no datasets were generated or analyzed during the current study.
